# Thoracic Aortic Rupture Post Cardiopulmonary Resuscitation in a Patient With Previous Thoracic Aneurysm Repair

**DOI:** 10.7759/cureus.41027

**Published:** 2023-06-27

**Authors:** Aniekeme S Etuk, Olanrewaju F Adeniran, Bernard I Nkwocha, Nformbuh Asangmbeng, Mina Jacob

**Affiliations:** 1 Internal Medicine, Thomas Hospital Infirmary Health, Fairhope, USA; 2 Internal Medicine, West Virginia University School of Medicine, Morgantown, USA; 3 Internal Medicine, University of Utah, Salt Lake City, USA; 4 Cardiology, Morehouse School of Medicine, Atlanta, USA; 5 Interventional/Structural Heart Cardiology, Mobile Infirmary Medical Center, Mobile, USA

**Keywords:** computed tomography scan, thoracic aortic aneurysm repair, cardiopulmonary resucitation, thoracic aortic rupture, thoracic aortic aneurysm, thoracic aortic dissection

## Abstract

Aortic dissection is characterized by a tear or rupture in the intimal layer of the aorta causing blood to flow between the layers of the arterial wall, thus separating them. While cardiopulmonary resuscitation (CPR) is a life-saving intervention, it can unintentionally contribute to the development or worsening of aortic dissection. The forceful chest compressions involved in CPR can put significant pressure on the fragile aortic wall, potentially leading to a tear or rupture. This highlights the delicate balance between life-saving measures and the potential risks they carry. Though studies have been done on the effects of CPR on the thoracic wall, few reports have studied the effects on the structures that lie in the thoracic cavity. The authors present a 63-year-old with a history of thoracic aneurysm repair who experienced a cardiac arrest while choking on food at home. The patient received CPR and a CT scan done thereafter revealed thoracic dissection and rupture. The patient received medical management in the Intensive Care Unit but eventually expired due to irreversible neurological damage. This highlights the importance of recognizing that CPR can pose a risk for aortic dissection and rupture, particularly in individuals with prior aortic repairs. It emphasizes the need for developing protocols to monitor patients who have undergone aneurysmal repair and adjusting CPR techniques to suit their specific needs. Additionally, further studies are needed to understand how often aortic complications occur after CPR and to provide guidance for follow-up care in patients who have had aortic repairs. By implementing these measures, we can improve outcomes and safety during resuscitation.

## Introduction

Survival following a cardiac arrest depends on effective cardiopulmonary resuscitation (CPR) [1]. Despite being a well-known procedure, CPR poses its own health risk. Injuries to the thoracic wall and cardiopulmonary system may cause significant morbidity and mortality from potentially reversible and, perhaps, unpredictable resuscitation outcomes [2]. While these are well documented in the literature, the incidence of thoracic aortic dissection or rupture (TAD/TAR) resulting from CPR is not well characterized, and no case has been reported in patients with previous aortic repairs [2]. We describe a case in which a CPR-associated aortic rupture was identified in a patient with previous aortic repair. The patient was medically managed in the ICU and, unfortunately, died from irreversible neurological damage following a prolonged downtime. We were compelled to report this case to establish that CPR following cardiac arrest is a risk factor for aortic dissection and rupture and to create awareness of the need to devise a protocol for monitoring aneurysmal repairs.

## Case presentation

A 63-year-old Caucasian male with three months history of thoracic endovascular aneurysm repair following a diagnosis of 6.6 cm thoracic aortic aneurysm was brought to the emergency room after developing a cardiac arrest on choking on a pork chop at home. His wife noticed he began coughing and fighting for breath in the middle of dinner and suddenly became cyanotic and unresponsive despite efforts at chest compressions. He had a past medical history of cerebrovascular accident (CVA) which occurred three years ago, leaving him with residual left hemiparesis, expressive aphasia, and difficulty clearing mucous secretions. He was a heavy smoker, with a 25-pack-year smoking history and significant alcohol consumption history.

**Figure 1:**
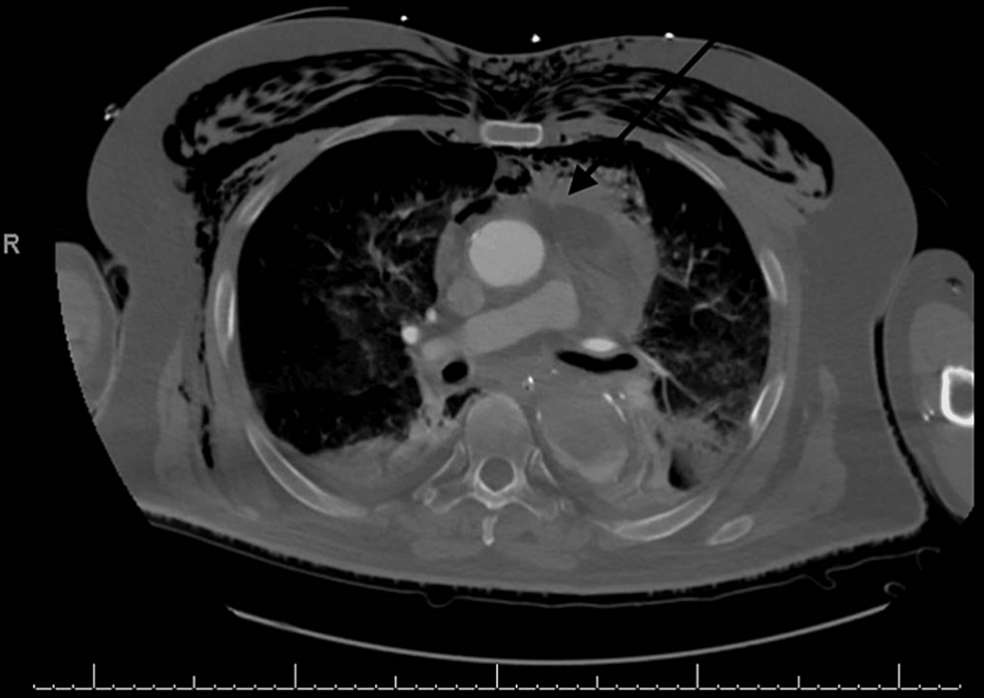
CT scan of the chest axial view showing hemopericardium; indicative of a potential aortic rupture (see black arrow)

**Figure 2:**
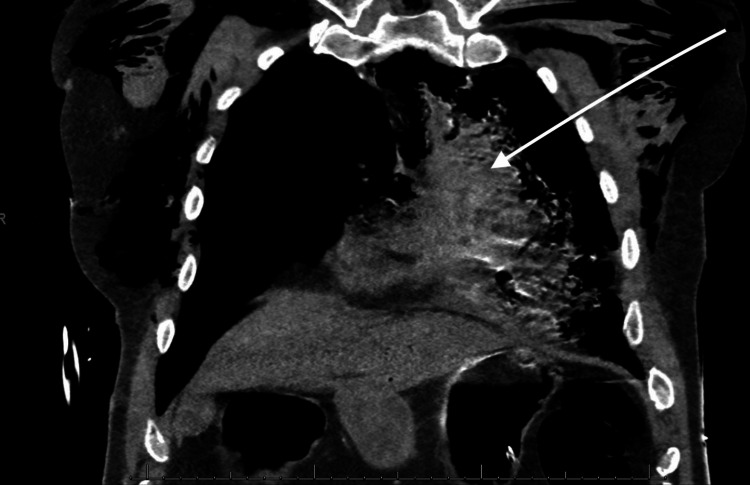
CT scan of the chest coronal view showing hemopericardium (see white arrow)

**Figure 3:**
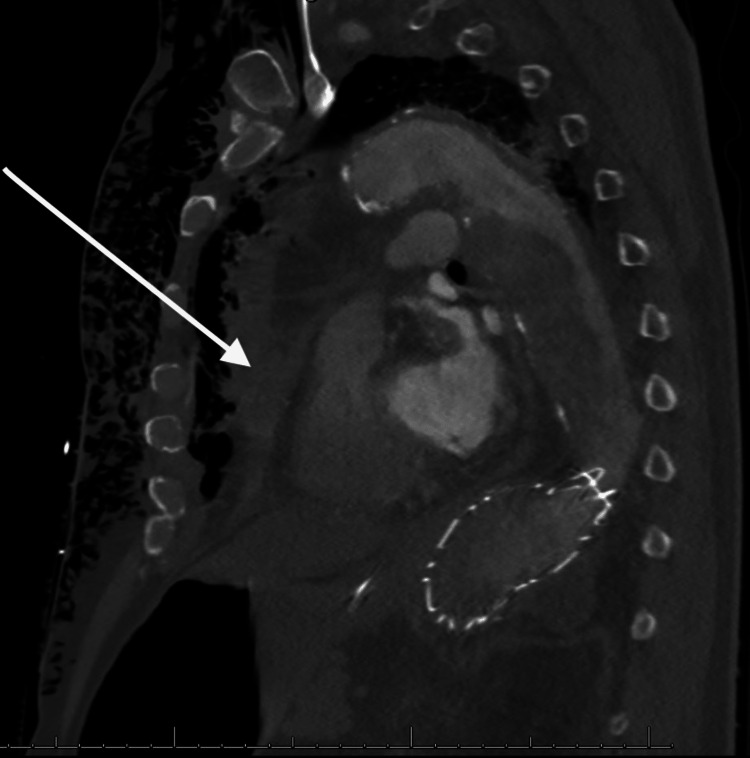
CT scan of the chest sagittal view showing hemopericardium (see white arrow)

Emergency Medical Service (EMS) arrived after about 15 mins of downtime to continue resuscitation, and he attained return of spontaneous circulation (ROSC) after 45 mins. At the emergency room, he was unresponsive, with a Glasgow Coma Scale (GCS) of 3/15, blood pressure of 96/70 mmHg, pulse rate of 140 bpm, and SPO_2_ of 92%. The complete metabolic panel and arterial blood gas revealed features of severe metabolic acidosis with a bicarbonate level of 12 mmol/L (24-28), pH of 6.9 (7.35-7.45), serum lactate of 26.1 mmol/L (0.5-1.6), and hyperkalemia of 5.9 mmol/L (3.6-5.2). A chest computerized tomography (CT) scan was done before transfer to the intensive care unit (ICU) and was concerning for a probable TAD with occlusion of the aorta at the descending aortic stent and a hemopericardium, indicating a rupture (Figures 1-3). It was difficult to appreciate the point of rupture in the imaging studies as the patient arrested during the process of scanning his chest.

He was managed in the ICU with IV Dopamine in D5W solution (400mg/250mg) at 2.5mcg/kg/min titrated and norepinephrine 4mg in 0.9% NaCl 250mL infusion at 5mcg/min titrated for blood pressure augmentation, and IV sodium bicarbonate 150mEq in D5W at 200mL/hr continuously, for the severe metabolic acidosis. Despite these interventions, the patient remained in a vegetative state with no improvement. The family was advised on the irreversible neurologic impairment that resulted from the prolonged downtime before the intervention, among other poor prognostic factors, such as the occluded descending thoracic aorta and irreversible metabolic acidosis. A collective decision was made to extubate and diagnose him as brain dead based on the presence of persistent coma, absence of brainstem reflexes and lack of ability to breathe independently.

## Discussion

CPR is a group of interventions performed to provide oxygenation and circulation to the body during cardiac arrest [3]. About 70% of out-of-hospital cardiac arrests occur at home, with no means of electrical defibrillation [4]. CPR offers a temporal measure until defibrillation or other definitive measures can be performed [4]. Conventional CPR, a manual procedure, combines chest compression with rescue breathing which can provide up to 33% of normal body cardiac output if done properly [4]. These resuscitative attempts require invasive iatrogenic manipulations of patients. However, external chest compressions may trigger blunt trauma due to the forceful compression of the sternum [1,5].

Thoracic aortic injury from blunt chest trauma is found in just about 1.5% to 2% of patients [5]. Before 2018, only one report of iatrogenic aortic injury from CPR was reported, by Juan et al., which described an intramural hematoma of the aorta found on a chest CT scan taken after CPR [6]. However, a direct relationship between aortic dissection/rupture and CPR could not be established as aortic integrity is rarely evaluated before CPR when cardiac arrests occur due to certain factors [6]. Chest radiography as a screening tool has a low sensitivity for thoracic aortic injuries. CT scan, with near 100% sensitivity, can diagnose other associated cardiothoracic injuries but often require patient transfer. Transesophageal echocardiography (TEE) can be used at the bedside in patients with hemodynamic instability but is largely operator dependent [7].

Our patient probably had a descending TAR at a previous aneurysm repair site three months after surgery. The rupture may have possibly been associated with the performance of CPR after choking or induced by the choking itself. Features correlating with blunt chest trauma, such as subcutaneous emphysema and hemopericardium were seen, but a causal factor could not be established as there was no CT scan or TEE done in the immediate premorbid state to assess the diameter and integrity of the thoracic aorta. Other possibilities could be that he had a slowly leaking aneurysm that was not detected early enough. Possible mechanisms of injury include pressure on the aorta between the displaced heart and the posterior thoracic wall during periodic heart compressions. Despite the lack of data, there is a theoretically increased risk for aortic injuries in already compromised aortas such as those with previous repairs [8].

The authors decided to report this case to raise awareness of the complications CPR poses in precipitating aortic rupture, especially in patients with previous aortic surgery, and to call for more studies on this subject matter to be undertaken to determine the true incidence of aortic dissection/rupture post-CPR.

## Conclusions

Despite being the mainstay for restoring circulation during cardiac arrest, CPR poses cardiopulmonary health risks, especially in patients with previous thoracic aortic repairs. Unfortunately, this has not been well illustrated in the literature to gain significant attention partly because there are no guidelines set in stone for monitoring patients with previous aortic repairs. Furthermore, investigating the state of the aorta before CPR poses a challenge due to time limitations, the sensitivity of the available imaging modalities, the accessibility of these techniques, and the availability of personnel. Concerning our patient, certain questions remained unanswered; could he have survived the cardiac arrest if we knew the state of his previous aneurysmal repair just before CPR to inform our decision? What adjustments to the standard CPR techniques should be made in such situations? The authors published this case report to draw necessary attention to the subject matter with the hope that a protocol will be developed to follow-up patients for features of dissection or rupture following the repair of an aortic aneurysm and call for a revision to the manual CPR techniques for these patients.
